# A Contractile Network of Interstitial Cells of Cajal in the Supratarsal Mueller's Smooth Muscle Fibers With Sparse Sympathetic Innervation

**Published:** 2012-02-15

**Authors:** Shunsuke Yuzuriha, Kiyoshi Matsuo, Ryokuya Ban, Shiharu Yano, Tetsuji Moriizumi

**Affiliations:** Departments of ^a^Plastic and Reconstructive Surgery; ^b^Anatomy, Shinshu University School of Medicine, Matsumoto, Japan.

## Abstract

**Background:** We previously reported that the supratarsal Mueller's muscle is innervated by both sympathetic efferent fibers and trigeminal proprioceptive afferent fibers, which function as mechanoreceptors-inducing reflexive contractions of both the levator and frontalis muscles. Controversy still persists regarding the role of the mechanoreceptors in Mueller's muscle; therefore, we clinically and histologically investigated Mueller's muscle. **Methods:** We evaluated the role of phenylephrine administration into the upper fornix in contraction of Mueller's smooth muscle fibers and how intraoperative stretching of Mueller's muscle alters the degree of eyelid retraction in 20 patients with aponeurotic blepharoptosis. In addition, we stained Mueller's muscle in 7 cadavers with antibodies against α-smooth muscle actin, S100, tyrosine hydroxylase, c-kit, and connexin 43. **Results:** Maximal eyelid retraction occurred approximately 3.8 minutes after administration of phenylephrine and prolonged eyelid retraction for at least 20 minutes after administration. Intraoperative stretching of Mueller's muscle increased eyelid retraction due to its reflexive contraction. The tyrosine hydroxylase antibody sparsely stained postganglionic sympathetic nerve fibers, whereas the S100 and c-kit antibodies densely stained the interstitial cells of Cajal (ICCs) among Mueller's smooth muscle fibers. A connexin 43 antibody failed to stain Mueller's muscle. **Conclusions:** A contractile network of ICCs may mediate neurotransmission within Mueller's multiunit smooth muscle fibers that are sparsely innervated by postganglionic sympathetic fibers. Interstitial cells of Cajal may also serve as mechanoreceptors that reflexively contract Mueller's smooth muscle fibers, forming intimate associations with intramuscular trigeminal proprioceptive fibers to induce reflexive contraction of the levator and frontalis muscles.

Mueller's smooth muscle fibers are serially located between the levator muscle fibers and the tarsus, under the levator aponeurosis; we have previously reported that Mueller's smooth muscle fibers are innervated by unmyelinated sympathetic efferent fibers, and furthermore, that the intramuscular connective tissues interspersed among the smooth muscle fibers are innervated by myelinated trigeminal proprioceptive afferent fibers (Fig 1a).^1^^,^^2^ The latter fibers function as mechanoreceptors, inducing reflexive contraction of 2 different eyelid-opening muscles, the levator and frontalis muscles. Voluntary contraction of the levator fast-twitch muscle fibers stretches the mechanoreceptors in Mueller's muscle to evoke trigeminal proprioception, thereby stimulating both the oculomotor neurons and the frontalis motoneurons to induce reflexive contraction of the levator and frontalis slow-twitch muscle fibers, respectively. This results in involuntary continuous lifting of the eyelid and eyebrow to maintain a visual field corresponding to changes in vertical gaze as a type of length servomechanism.^3^^-^9

Aponeurotic blepharoptosis is caused by disinsertion of the levator aponeurosis from the tarsus and elongated attenuation of the levator aponeurosis and underlying Mueller's muscle (Figs 1b and 2a).^10^^-^12 During eyelid opening in patients with aponeurotic blepharoptosis, the retractile force of the levator muscle is transmitted to the tarsus via the sympathetically innervated Mueller's muscle instead of the aponeurosis. It has been empirically noted that the eye will open quite normally despite total disconnection of the aponeurosis, as long as there is a normally functioning Mueller's muscle.^13^ Therefore, in patients with aponeurotic blepharoptosis, stretching of Mueller's muscle must induce contraction of Mueller's smooth muscle fibers for transmission of the retractile force from the levator muscle to the tarsus.

As controversy persists around the identity and physiological roles of the mechanoreceptor in Mueller's muscle, we sought to clinically and histologically investigate Mueller's muscle.

## METHODS

Phenylephrine (an α_1_-selective agonist) was administered into the upper fornix to contract the partial Mueller's muscle in each of 20 patients (15 women and 5 men; 40.9 ± 5.2 years old) with aponeurotic blepharoptosis. Patients were made to lie in a supine position, raise their chin, and gaze downward: the upper eyelid on the side of the dominant eye was pinched for 60 seconds to detach it from the globe and create a space in the upper fornix. Two to 3 drops of 5% phenylephrine were administered into the space, and the phenylephrine was retained in this position by gravity to exclusively stimulate the unilateral posterior Mueller's smooth muscle fibers that face the conjunctiva palpebrae. Changes in the distance between the upper eyelid margin and the line between the medial and lateral canthi were measured as upper eyelid retraction distance (UERD). Measurements were taken before and subsequently 1, 2, 3, 4, 5, 10, and 20 minutes after administering phenylephrine using digital photographs on primary gaze with a 10-mm square scale (Casmatch; Dai Nippon Printing Co, Ltd, Tokyo, Japan). The average time of maximal eyelid retraction induced after phenylephrine instillation was calculated.

During aponeurotic blepharoptosis surgery, UERDs were measured upon eyelid opening after unilateral reinsertion of the levator aponeurosis to the tarsus to unilaterally desensitize the mechanoreceptors in Mueller's muscle in the 20 patients. Subsequently, the bilateral tarsi were pulled caudally as far as possible without inducing pain for 5 seconds, and within a second of eyelid opening, the UERDs were measured again. All intraoperative measurements of the UERD were made on the basis of the horizontal corneal diameter and referenced to the preoperative digital photographs with a 10-mm square scale. Before and after caudal pulling of the bilateral tarsi, changes in the UERDs of the bilateral eyelids were assessed for statistical differences between the eyelids with and without the reinsertion.

For histological investigation of Mueller's muscle, 7 specimens of unilateral Mueller's muscle were obtained from 7 Japanese cadavers (3 women and 4 men; age: 82.6 ± 6.2 years). The specimens were fixed in 10% buffered formalin and processed for routine paraffin embedding. Specimens were serially sliced along the horizontal plane (8- to 10-µm thickness). The serial sections were processed for immunohistochemical staining with the avidin-biotin-peroxidase complex method. Sections were microwaved and individual sections were incubated with primary antibodies against α-smooth muscle actin (DAKO Japan Co, Ltd, Kyoto, Japan), S100 (DAKO Japan Co, Ltd, Kyoto, Japan), tyrosine hydroxylase (PROTOS BIOTECH Co, New York), c-kit (DAKO Japan Co, Ltd., Kyoto, Japan), and connexin 43 (Spring Bioscience Co., Pleasanton, Calif); antibodies were diluted to 1:100-400 and incubated with sections at 25°C for 12 to 24 hours, followed by 2 consecutive incubations with secondary antibodies and streptavidin conjugated to horseradish peroxidase (DAKO Japan Co, Ltd). Final visualization of all sections was obtained by adding 0.01% diaminobenzidine plus 0.0015% H_2_O_2_ in 0.05 M Tris-HCL buffer, pH 7.6. In addition, toluidine blue staining was used to differentiate ICCs from mast cells with metachromasia.

Five cross-sectional areas of Mueller's muscle were randomly selected from the 7 specimens. The average number of postganglionic sympathetic nerve fibers stained positive for tyrosine hydroxylase and ICCs were counted in a microscopic field 260 µm high × 340 µm wide at 400× magnification (BX50 microscope, Olympus, Tokyo, Japan).

Data were statistically analyzed using Friedman and paired *t* tests. A *P* value less than .05 was considered to indicate a significant difference. All data are represented as mean ± SD.

## RESULTS

Maximal eyelid retraction due to pharmacological contraction of Mueller's smooth muscle fibers took 3.8 ± 0.8 minutes; however, initial effects were evident at 1 minute (Figs 2a-c). The induced eyelid retraction was still evident at 20 minutes after administration of phenylephrine, albeit with a gradual decline (Fig 2d). Although the eyelid retraction increased, the ipsilateral eyebrow height decreased inversely (Figs 2b-d).

Prior to caudal pulling of the bilateral tarsi, the UERDs of the eyelids without reinsertion (3.6 ± 0.6 mm) were significantly smaller than those with reinsertion (5.3 ± 1.0 mm, *P* < .0001) (Figs 3a, d, f, and h). After caudal pulling of the bilateral tarsi for 5 seconds, the UERDs of the eyelids without reinsertion (4.8 ± 0.8 mm) remained significantly smaller than those with reinsertion (5.9 ± 0.9 mm, *P* = .0003) (Figs 3c, e, g, and i). In addition, the UERDs of the eyelids with reinsertion increased significantly after caudal pulling of the bilateral tarsi (5.9 ± 0.9 mm vs 5.3 ± 1.0 mm, *P* = .0001) (Figs 3h and i). The UERDs of the eyelids without reinsertion also increased significantly after caudal pulling of the bilateral tarsi (4.8 ± 0.8 mm vs 3.6 ± 0.6 mm, *P* < .0001) (Figs 3h and i). The increases in the UERDs of the eyelids without reinsertion after caudal pulling (1.3 ± 0.4 mm) were significantly greater than those with reinsertion (0.6 ± 0.3 mm, *P* = .0001) (Fig 3j).

Mueller's muscle fibers were independently stained with α-smooth muscle actin antibody (Fig 4a). Schwann cells of the myelinated proprioceptive nerve fibers were densely stained with an S100 antibody (Fig 4b).^1^^,^^2^ A tyrosine hydroxylase antibody only sparsely stained 4.7 ± 1.1 unmyelinated postganglionic sympathetic nerve fibers (Fig 4c); the c-kit antibody, however, stained 7.3 ± 1.5 interstitial cells of Cajal (ICCs; Fig 4d) and 6.3 ± 1.2 mast cells (Fig 4f) in a microscopic field 260 µm high × 340 µm wide at 400× magnification. Mast cells were identified by staining with toluidine blue. The average number of the ICCs was significantly greater than the number of the sympathetic fibers (*P* = .018). The gap junctions in Mueller's smooth muscle fibers, however, were not clearly stained by a connexin 43 antibody (Fig 4e); as a control, the gap junctions in the human cardiac muscle were well stained with a connexin 43 antibody (Fig 4e, insert).

## DISCUSSION

Pharmacological contraction of the posterior Mueller's smooth muscle fibers by administration of phenylephrine to the superior fornix gradually propagated to the anterior Mueller's smooth muscle fibers, and maximal eyelid retraction occurred at approximately 3.8 minutes; the whole Mueller's muscle fibers contracted sufficiently to retract the eyelid to a kind of syncytium. Pharmacological induction of eyelid retraction lasted more than 20 minutes and, however, gradually declined after the first 3.8 minutes. Pharmacological contraction of Mueller's smooth muscle fibers desensitized the mechanoreceptors among them and prevented stretching by contraction of the levator muscle; this increased ipsilateral eyelid retraction but decreased reflexive contraction of the ipsilateral levator and frontalis slow-twitch muscle fibers, resulting in decreased retraction of the contralateral eyelid (Hering's law of equal innervation to the levator muscles) and ipsilateral eyebrow height (Fig 2c). We have previously reported that pharmacological contraction of canine Mueller's smooth muscle fibers by retrograde administration of α_1*A*_-adrenoceptor agonist to the drained vein of Mueller's muscle lasted for 92 minutes.^14^ Other studies have demonstrated that phenylephrine injected into the femoral vein induces sustained contraction of Mueller's muscle in anesthetized rats.^15^ These findings suggest the involvement of a structure that mediates neurotransmission among Mueller's smooth muscle fibers and sustains their prolonged contraction.

Reinsertion of the levator aponeurosis to the tarsus resulted in significantly decreased stretching of the mechanoreceptors in Mueller's muscle upon caudal pulling of the tarsi (Fig 3b). The reflexive contraction of the levator muscle induced by caudal pulling of the tarsi was not maintained after cessation of the pulling force because of a stretch reflex in the skeletal muscle. Therefore, in the eyelid without reinsertion, we interpret the significant increase in the UERD after the cessation of caudal pulling of the tarsi as a result of prolonged reflexive contraction of Mueller's muscle. The mean maximum total excursion of the upper eyelid due to the action of Mueller's muscle was reported to be 3.0 mm, with 1.5 mm upward and 1.5 mm downward displacement from its tonic position.^16^ In our study, the mean increases in UERD of 1.3 mm due to the caudal pulling was consistent with the published report.

**Figure 1:**
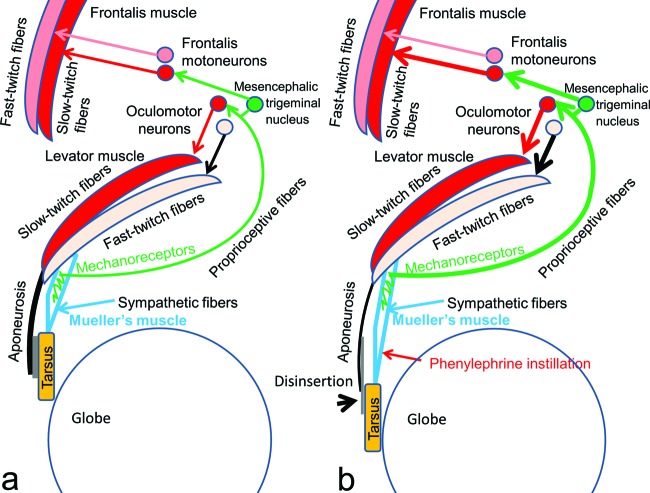
A neurophysiological schema involuntarily maintains an adequate visual field by reflexive contraction of the levator and frontalis slow-twitch fibers during changes in vertical gaze as a type of length servomechanism. (a) Normal condition. (b) Aponeurotic blepharoptosis. Reflexive contraction of the levator and frontalis slow-twitch muscle fibers are increased by enhanced voluntary contraction of the levator fast-twitch muscle fibers.

**Figure 2:**
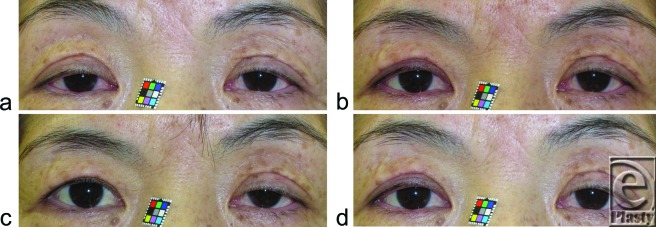
Changes in the upper eyelid retraction distance (UERD) before and after phenylephrine-mediated contraction of the posterior Mueller's muscle that faces the conjunctiva palpebrae. (a) A 58-year-old woman with aponeurotic blepharoptosis prior to administration of phenylephrine on the right side. (b) One minute, (c) 4 minutes, and (d) 20 minutes after administration of phenylephrine.

**Figure 3:**
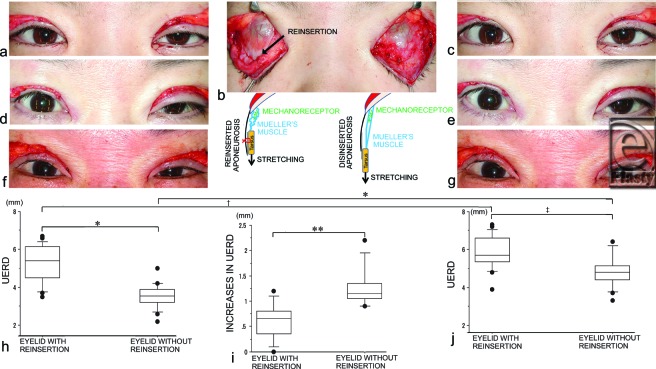
Changes in the upper eyelid retraction distance (UERD) before and after intraoperative stretching of Mueller's muscle. (a) Eyelid opening after unilateral reinsertion of the levator aponeurosis to the tarsus in a 28-year-old female patient. (b) Caudal pulling of the bilateral tarsi to stretch the mechanoreceptors in Mueller's muscle (note that the right aponeurosis was reinserted to the tarsus with 3 stitches). (c) Eyelid opening after caudal pulling. (d) Eyelid opening after unilateral reinsertion and (e) after caudal pulling in a 35-year-old female patient. (f) Eyelid opening after unilateral reinsertion and (g) after caudal pulling in a 60-year-old female patient. (h) The UERDs of the eyelids with and without reinsertion. (i) The increase in UERDs of the eyelids due to caudal pulling with and without reinsertion. (j) The UERDs of the eyelids with and without reinsertion 1 second after stretching of the mechanoreceptors in Mueller's muscle. (*P < .0001; †*P* = .0001; ‡*P* = .0003.)

**Figure 4:**
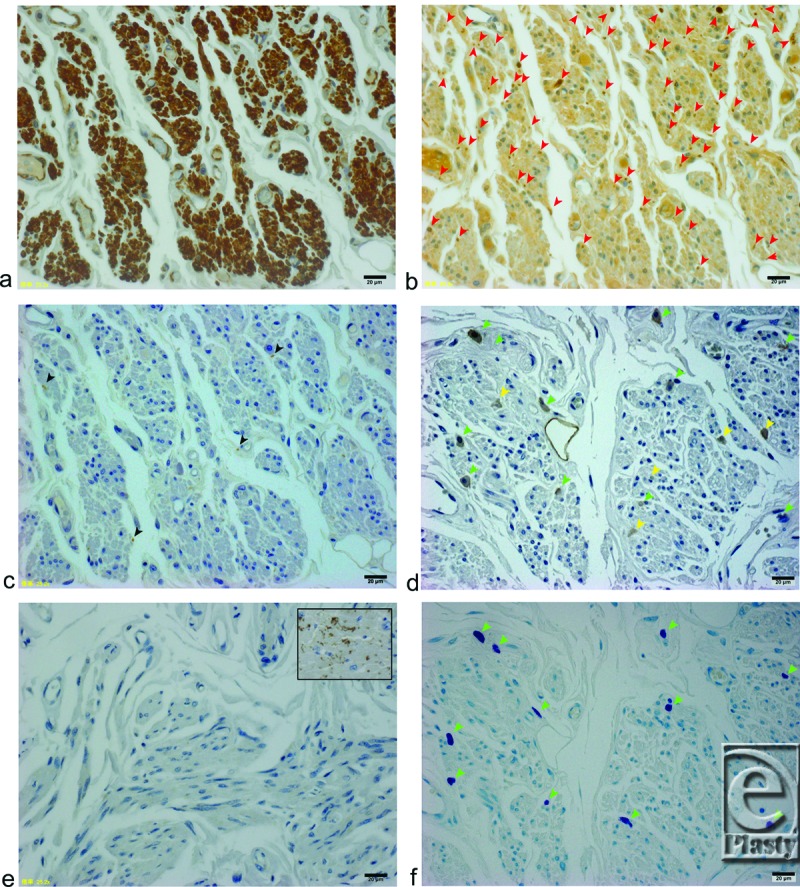
Histological analysis of serial sections of Mueller's smooth muscle fibers in a microscopic field 260 µm high × 340 µm wide at 400× magnification. (Insets: scale bars = 20 µm) (*a*) α-Smooth muscle actin antibody staining. Smooth muscle fibers of Mueller's muscle are stained brown. (b) S100 antibody staining. Myelinated nerve fibers are stained dark brown (red arrow heads). (c) Tyrosine hydroxylase antibody staining. Unmyelinated postganglionic sympathetic nerve fibers are stained brown (black arrowheads). (d) C-kit antibody staining. Both ICCs (yellow arrowheads) and mast cells (green arrowheads) are stained brown. (e) Connexin 43 antibody staining. Inset shows a positive control, and human cardiac muscle fibers are stained brown. (f) Toluidine blue staining. Mast cells are stained purple (green arrowheads).

It is plausible that the reflexive contraction of Mueller's muscle is induced by stretching of Mueller's muscle and this assumption has been supported in several studies^4^^,^^17^^-^20; one study demonstrates that upgaze improves ptosis whereas downgaze worsens ptosis in acquired blepharoptosis such as aponeurotic blepharoptosis. In addition, upgaze increases stretching of Mueller's muscle, thereby resulting in its reflexive contraction, whereas downgaze decreases stretching of Mueller's muscle, thereby relaxing the muscle. The muscle bellies of the levator and Mueller's muscles function as a single serial muscle unit in an aponeurotic blepharoptosis (Fig 1b) 21; therefore, it is likely that the degree of reflexive contraction of the levator muscle is exaggerated by the degree of reflexive contraction of Mueller's muscle in patients with aponeurotic blepharoptosis.

Histologically, distributions of unmyelinated postganglionic sympathetic nerve fibers (as stained by the tyrosine hydroxylase antibody) were more sparse than expected. On the contrary, ICCs (stained by c-kit antibody) were densely stained among Mueller's smooth muscle fibers. Mast cells, which were also stained by c-kit antibody, were differentiated from ICCs with metachromasia by toluidine blue staining. Myelinated trigeminal nerve fibers, which correspond to the proprioceptive nerve fibers, were also densely stained by S100 antibody as we have previously reported.^1^^,^^2^ Although the connexin 43 antibody is known to stain the gap junctions in vascular smooth muscle fibers, myoepithelial smooth muscle fibers, and ICCs in the bladder and gastrointestinal tract,^22^^-^25 it stained neither Mueller's smooth muscle fibers nor ICCs. Therefore, other connexins such as connexin 26, 32, etc, may exist to connect the ICCs and Mueller's smooth muscle fibers.

**Figure 5:**
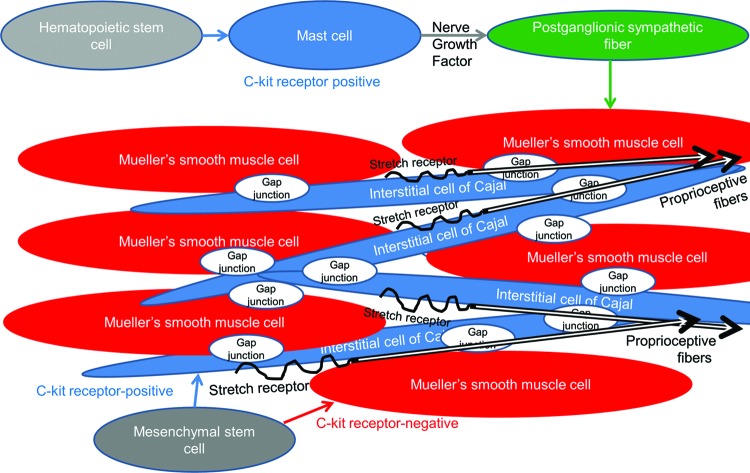
Hypothetical relationships between Mueller's smooth muscle fibers, ICCs, post-ganglionic sympathetic efferent fibers, trigeminal proprioceptive afferent fibers, and mast cells.

Previous reports have established several functions mediated by ICCs, including (i) their role as pacemakers that actively propagate slow electrical waves in gastrointestinal muscles, resulting in their peristalsis; (ii) mediation of both inhibitory and excitatory motor neurotransmission; (iii) their role as nonneural stretch receptors in gastrointestinal muscles and the bladder, affecting both smooth muscle excitability and slow wave frequency; and finally (iv) their ability to form intimate associations with the intramuscular terminals of vagal afferents, potentially conferring a role in afferent signaling.^23^^,^^24^^,^^26^^-^37 Phenylephrine-induced contraction of posterior Mueller's smooth muscle fibers has been shown to gradually propagate to anterior Mueller's smooth muscle fibers. As a consequence, it is likely that a contractile network of ICCs may mediate neurotransmission between Mueller's multiunit smooth muscles fibers, which are sparsely innervated by postganglionic sympathetic fibers (Fig 5). In addition, intraoperative stretching of Mueller's muscle results in their reflexive contraction. Interstitial cells of Cajal may also serve as mechanoreceptors that cause their reflexive contraction (Fig 5). Interstitial cells of Cajal may also form intimate associations with intramuscular trigeminal proprioceptive fibers (stained densely by the S100 antibody), which function as mechanoreceptors, inducing reflexive contraction of the levator and frontalis muscles (Fig 5). Both c-kit-positive ICCs and c-kit-negative Mueller's smooth muscle fibers arise from mesenchymal stem cells.^38^^-^41 The major difference between ICCs and Mueller's smooth muscle fibers is the presence or absence of the c-kit gene, the protooncogene encoding a receptor tyrosine kinase,^42^ which dictates cellular functions such as the mediation of neurotransmission and contraction.

Mast cells are components of connective tissue in many regions of the body. Their functions are poorly understood; however, they are believed to release nerve growth factor^43^ as postganglionic sympathetic nerve fibers are unmyelinated and lack Schwan cells, which are normally required to release nerve growth factor in myelinated nerve fibers.^44^

## CONCLUSIONS

Mechanoreceptor stretching in Mueller's muscle (which associates with both the intramuscular terminal of trigeminal proprioceptive afferents and ICCs) may induce reflexive contraction of the levator and frontalis slow-twitch muscle fibers as well as Mueller's smooth muscle fibers. Further studies are required to confirm the presence of the connexin and stretch receptors in Mueller's smooth muscle fibers.

An additional hypothesis suggests that stretching of the mechanoreceptors in Mueller's muscle increases the sympathetic tone resulting in its contraction via the trigeminal proprioceptive afferent fibers and the sympathetic efferent fibers.

## Acknowledgments

This work was supported in part by a Grant-in-Aid for Scientific Research (C) from the Japan Society for the Promotion of Science JSPA) (KAKENHI; No. 21592284).

None of the authors have any commercial associations or financial interests that may pose or create a conflict of interest with the information presented in this article.
